# Photonic Voltage Transducer with Lightning Impulse Protection for Distributed Monitoring of MV Networks

**DOI:** 10.3390/s20174830

**Published:** 2020-08-26

**Authors:** Grzegorz Fusiek, Pawel Niewczas

**Affiliations:** Department of Electronic and Electrical Engineering, University of Strathclyde, Glasgow G1 1XW, UK; p.niewczas@strath.ac.uk

**Keywords:** optical voltage sensors, fibre Bragg gratings, piezoelectric transducer, power system instrumentation, medium voltage networks, distributed sensing

## Abstract

The design, construction and characterization of a photonic voltage transducer with a lightning impulse protection for distributed measurements on medium voltage (MV) networks (11 kV) was presented in this paper. The sensor prototype, comprising a combination of a piezoelectric transducer and a fibre Bragg grating (FBG) as a core optical sensing element, and a dedicated lightning protection device comprising a set of reactive components, was evaluated through laboratory testing and its performance was assessed based on the accuracy requirements specified by the relevant industry standards. It was demonstrated that the sensor has the potential to meet the accuracy requirements for the 3P protection and 0.2 metering classes specified by the IEC 60044-7. The device successfully underwent lightning impulse withstand tests, satisfying the safety requirements applicable to 11 kV networks as specified by the standard. The usage of an FBG as a photonic sensing component enables the multiplexing of multiple such sensors to provide the distributed measurement of voltage along a power network.

## 1. Introduction

Electricity distribution networks are a key element of the wide-area power system. The distribution grid utilizes overhead lines, underground cables and step-down transformers to distribute the electrical power to the primary and secondary customers. In Europe, the distribution system voltage levels are between 0.4 kV and 225 kV, and in the UK distribution network, typical voltage levels are 0.4/11/33/132 kV [1]. 

Among the various problems related to the electrical network functionality and stability, there are different types of faults that can be either temporary, causing no damage to the electrical equipment and leading to temporary power outages, or permanent, causing the failure of the system equipment and leading to blackouts with durations sometimes reaching several hours [2]. Temporary faults constitute 50–90% of faults in overhead distribution systems, however, after a short delay (below 3 min) the network can operate normally [2,3]. Around 50% of faults are caused by abnormal weather conditions with lightning strikes being the reason for nearly 10% of faults. Normally, lightning events cause temporary faults on distribution networks with less than 20% of lightning strikes causing permanent damage [2,3,4]. In the majority of cases, when the lightning event occurs, the equipment insulation can be damaged due to the high current surge in the line that generates large voltage impulses—often leading to flashovers unless the equipment was protected with surge arresters [2] or is furnished with sufficient insulation that meets the relevant industry standards [5]. 

Wide-area monitoring, protection and control (WAMPAC) systems, facilitating the increased network visibility, improved the reaction time to the network demands and disturbances, and improvements to network reliability and security, play an inevitable role in the prevention of blackouts. Wide-area disturbance protection and control require distributed voltage and current measuring instrumentation, as well as fast and reliable communication links in order to provide the knowledge of the complete state of the power system over large geographical areas in real-time [6,7]. As part of the developing WAMPAC technologies, novel sensor systems capable of providing distributed voltage and current measurements while remaining cost-competitive with the current technology are desired.

To enable access to multiple, remote, distributed, passive current and voltage measurements over long distances that can be applicable to a wide range of metering and protection applications, the concept of a photonic sensors suite utilizing fibre Bragg grating (FBG) sensors and piezoelectric transducers was proposed by the authors [8,9,10,11]. This included variants of the core technology—from using low-voltage piezoelectric stacks to measure the output of a conventional CT or Rogowski coil for current measurement [8,9,12], through to using the same approach for sensing the secondary voltage of a capacitor divider to achieve voltage measurement at 132 kV [11]—to direct the voltage measurement at medium voltage by using larger piezoelectric transducers that are capable of being exposed to multi-kV-level voltages [10]. The latter device has the benefit of using a “hard” piezoelectric transducer that generally has a better stability and long-term performance as opposed to the “soft” piezoelectric transducers [13,14]. However, in order to meet the safety requirements stipulated by the IEC standard, the devices are also required to undergo successful lightning impulse tests. This can be a challenge for piezoelectric transducers that directly sense the primary voltage. From the initial tests, it was apparent that the first prototypes based on the direct voltage measurement approach were not capable of withstanding the standard lightning tests.

In this paper, we concentrate on the design, construction and evaluation of the improved prototype photonic voltage transducer (PVT) for medium voltage networks that are based on the direct application of primary voltage to the piezoelectric transducer and is capable of withstanding the standard lightning impulse test. We also assess its potential to comply with metering and protection class requirements according to IEC 60044-7 for Electronic Voltage Transformers as the most appropriate standard [5]. Details of the selection of components and packaging used in the construction of the sensor are proposed in order to ensure compliance with the safety requirements applicable to 11 kV networks. The device performance is verified by carrying lightning impulse withstand tests as defined by IEC 60044-7. 

## 2. Photonic Voltage Transducer Design and Construction

### 2.1. Design Requirements

The photonic voltage transducer is to be connected between one line of an 11 kV system and earth, and hence, its rated voltage is 6.35 kV (9 kV peak) [10]. For a primary rated voltage of 6.35 kV, the relevant rated voltage factors are 1.2 and 1.5, which are applicable to measurements between phase and earth on a continuous and 30 s duration basis, respectively [10]. Additionally, the device must withstand the rated power frequency for 60 s and 30 positive and negative impulses of the rated lightning impulse voltage levels as summarized in Table 1.

Since the sensor is aimed at being compliant with 3P protection and 0.2 metering classes, its voltage measurement errors at the rated frequency must be below the limits specified in Table 2 and Table 3, respectively [5].

It should be noted that for metering class, the voltage and phase errors at the rated frequency should not exceed the values specified in Table 3. at any voltage between 80% and 120% of the rated voltage, *U*_pn_ [5]. 

### 2.2. Piezoelectric Transduser Lightning Impulse Testing

**Figure 1:**
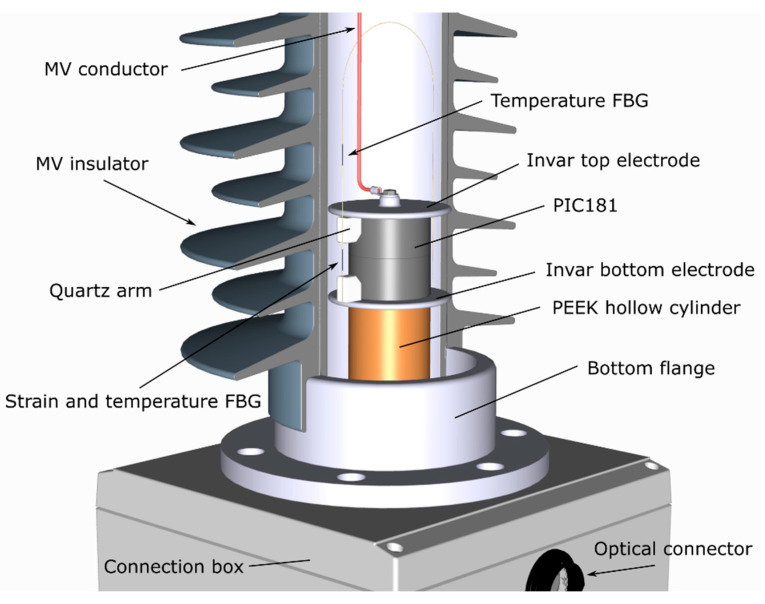
Construction of the first prototype photonic voltage transducer for medium voltage (MV) networks.

In the originally proposed design [10], shown in Figure 1, an FBG sensor was suspended between two quartz arms bridging a cylindrical block of PIC181 piezoelectric material, having a diameter of 40 mm and a thickness of 40 mm (2 × 20 mm thick cylinders). The FBG was attached to the arms with a UV epoxy and the attachment method was aimed at providing strain amplification. Two electrodes of 60 mm in diameter were used to ensure a more favourable electric field distribution around the piezoelectric transducer, moving the high fields away from the transducer. A flexible medium voltage (MV) conductor with a 2 mm diameter was used to connect the line voltage to the top electrode. The bottom electrode was connected to the earth directly due to the nature of the construction. The assembly was placed on top of a polyether ether ketone (PEEK) hollow cylinder to provide room for securing fibres and enable the connection of the bottom electrode to the ground. To enable DC measurement, the sensor was equipped with an additional temperature-sensing FBG to enable temperature compensation in this mode of operation. The sensor was placed in a hollow core composite insulator and potted in a dielectric gel, offering a dielectric strength of 23 kV/mm, to provide additional protection against electrical discharge and external vibrations [10].

**Figure 2:**
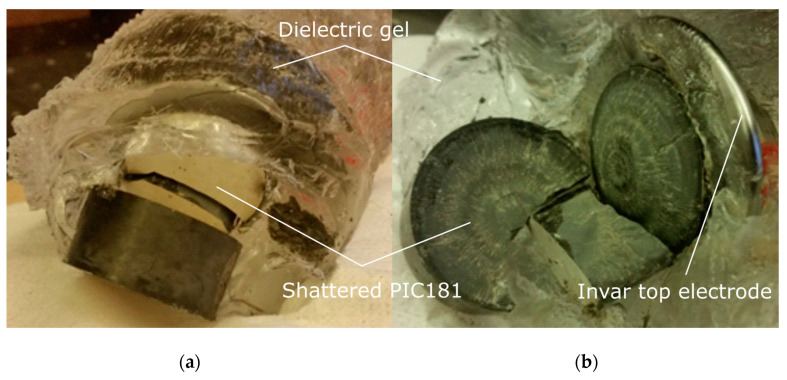
Failure of the first prototype MV photonic voltage transducer (PVT) after a series of lightning impulses that caused excessive accelerations, resulting in internal forces that exceeded the material’s mechanical strength. The picture illustrates the shattered piezoelectric transducer, taken out from the disassembled sensor unit, still partially covered with dielectric gel. The piezoelectric stack broken apart (**a**) and shattered piezoelectric component with a visible crater (**b**).

In order to allow for the installation on an 11 kV overhead line, a PVT prototype was tested against 75 kV lightning impulses of both polarities according to the IEC 60044-7 standard. It successfully passed 15 positive impulses, but was severely damaged after the fourth negative impulse, as shown in Figure 2.

For a PIC181 cylinder (Physik Instrumente Ltd. (Bedford, UK)) of the 40 mm thickness (note that two 20 mm cylinders were used to form a 40 mm stack), the maximum electric field of nearly 1 kV/mm can be expected at a power frequency withstand voltage of 28 kV (rms) and around 0.2 kV/mm at a rated voltage of 6.35 kV (rms). The electric field in the material when the sensor is exposed to the lightning impulse of 75 kV (peak) is estimated at 1.8 kV/mm. 

It should be noted that at the appropriate insulation of the transducer, it might survive even higher fields; especially those created by short time duration voltage pulses, as in the case of the lightning impulse withstand voltage considered here. It should be borne in mind, however, that the performance of piezoelectric materials over time is influenced by operating temperature, voltage levels, and humidity. Therefore, the maximum electric field expected during the piezoelectric transducer operation should be selected with a sufficient margin in the transducer design process. 

Since the thickness of the transducer was selected to be 40 mm to ensure that the electric field in the material was well below 2.5 kV/mm at 75 kV [15], the failure of the component must have been caused by the excessive tensile stresses in the material when subjected to excessive accelerations resulting from positive lighting impulses.

According to the manufacturer of PIC181 [15], the maximum static compressive strength for the material was approximately 600 MPa. To avoid depolarization or any other damage to the material, the components should not be compressed to more than 20–30% of this value. This is equivalent to 120–180 MPa. 

Even more stringent requirements are for operating the component in the tensile mode. Tensile forces are critical for the piezoelectric materials and should be avoided or minimized by applying a preload, if possible. According to the manufacturer, the maximum tensile stress in the material should not be greater than 10% of the compressive stress limit mentioned above, indicating a range of 12–18 MPa. It should be noted that the higher the applied negative voltage and the higher the frequency, the more tensile stress is generated in the material. 

The manufacturer also stated that even though the material can withstand approximately 600 MPa in compression, the piezoelectric materials may depolarize partly at loads of 50 MPa and higher, and at above 100 MPa they may depolarize completely. As the fracture mechanism of such poly-crystalline materials follow a specific fracture mechanism that is very similar to a Weibull distribution, the manufacturer does not specifically define the maximum allowed stress for their materials. However, they recommend completely preventing tensile impacts if possible, or compensate with a preload as “only 5–10 MPa can be tolerated at a low number of impact cycles with limited risk of failing.” [15]. 

For this reason, a 10 MPa tensile stress was assumed as the limit that should not be exceeded during the PVT operation and lightning impulse events.

### 2.3. Lightning Impulse Attenuator Requirements

As discussed in the previous section, due to the rapid change in voltage during a lightning impulse event, the piezoelectric component will undergo a shock that will result in large mechanical oscillations. Stress in the material will breach the recommended tensile stress limits for the material, potentially resulting in physical damage. One practical solution to overcome this problem is to use an electrical filter connected in series with the piezoelectric transducer to sufficiently attenuate the lightning impulse to avoid damage to the piezoelectric component, but allowing to transmit 50 Hz signals without significant change—in order to meet the relevant amplitude and phase error requirements as specified in the IEC standards. The circuit analysis was performed in MATLAB in frequency and time domains.

### 2.4. Frequency and Step Response Simulations

**Figure 3:**
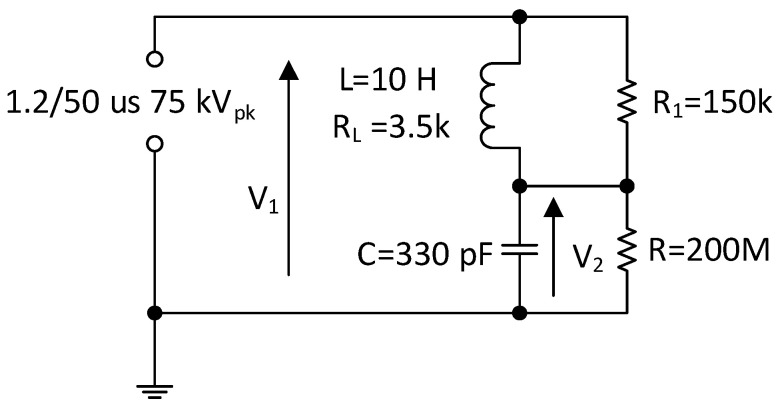
Equivalent circuit diagram of the piezoelectric stack (C and R) and lightning impulse attenuator, L, R_L_ and R_1_.

To evaluate the theoretical performance of the PVT and to estimate the expected amplitude and phase responses, the Bode analysis was performed in MATLAB. For the operation of the stack below the resonant frequency, the component can be treated as a capacitor and is represented by RC components as shown in Figure 3, where R is the stack resistance, and C is its capacitance.

To block the high frequency signals that could damage the piezoelectric component, it was proposed that an RL filter would be added in series with the piezoelectric transducer. A diagram of the considered circuit is shown in Figure 3. 

For the circuit above, the Laplace transfer function describing the relation between the input voltage (V_1_) and the output voltage (V_2_) of the system is given by

(1) $$\mathrm{TF}\left(s\right)\;=\;\frac{V_{2}\left(s\right)}{V_{1}\left(s\right)}\;=\;\frac{R_{1}\;+{\;R}_{L}\;+\;\mathrm{sL}}{s^{2}{\mathrm{LCR}}_{1}\;+\;s\left({\mathrm{CR}}_{L}R_{1}\;+\;L\frac{R_{1}}{R}\;+\;L\right)\;+\;\frac{R_{1}R_{L}}{R}\;+{\;R}_{1}\;+{\;R}_{L}}$$

The resistance of the piezoelectric component was assumed based on the data available from the manufacturer for PIC252, showing the material resistance as a function of time at 200 °C. Although the resistance decreases with aging, it is well above 120 MΩ even when held at 200 °C for over 1000 h [15]. Therefore, 200 MΩ resistance of the PIC181 was assumed for its R representation at room temperature. The capacitance was calculated from the PIC181 dimensions and specifications, and confirmed by capacitance measurements.

Based on (1), several MATLAB simulations were run with different values for the inductor parameters (L, R_L_) and the parallel resistance (R_1_) to analyse the influence of these components on the magnitude and phase response of the circuit. The piezoelectric RC values remained constant during the simulations.

**Figure 4:**
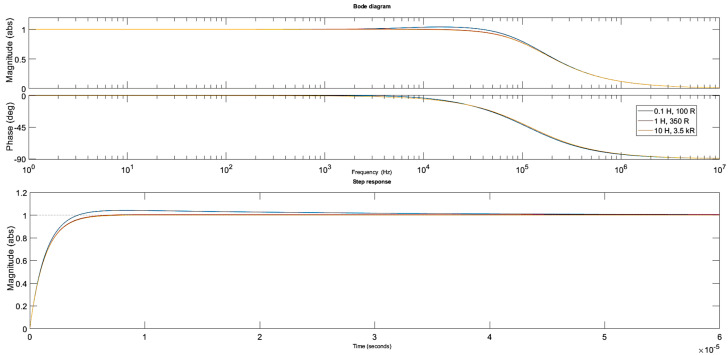
Bode and step response plots for the different inductor parameters. The parallel resistor R_1_ and the piezo stack settings remained constant.

Firstly, the parallel resistance R_1_ was arbitrarily set to 4 kΩ while the inductor parameters L and R_L_ were estimated as described in Section 2.7.1 and specified as follows: 0.1 H, 100 Ω; 1 H, 350 Ω; 10 H, 3.5 kΩ. The simulations were then run for these three cases. The Bode and step response plots are shown in Figure 4.

Clearly, the influence of the inductor parameters on the circuit response is limited when a relatively small resistance of the parallel resistor is used. The 3 dB bandwidth of the circuit was approximately 120 kHz and the circuit response to the step voltage was not reduced significantly with a rise time of nearly 3 µs. An increase in the parallel resistance is required to limit the bandwidth further. 

**Figure 5:**
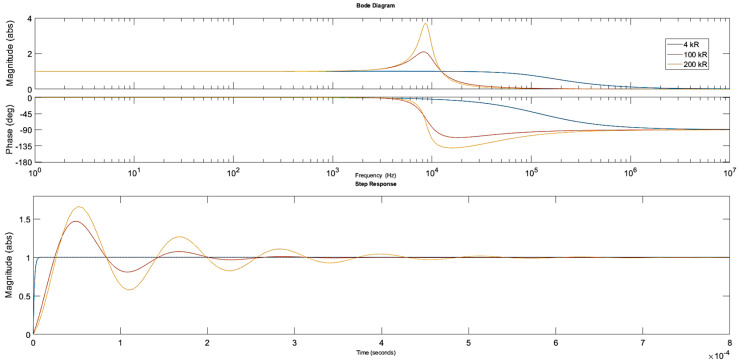
Bode and step response plots for the different parallel resistance values. The inductor (1 H, 350 Ω) and the piezo stack settings remained constant.

The second set of simulations involved changing the parallel resistance values while the inductor and the piezoelectric component parameters remained unchanged. The simulation results shown in Figure 5 are for a 1 H inductance and for the following settings of the parallel resistance R_1_: 4 kΩ, 100 kΩ, 200 kΩ.

It can be clearly seen that larger R_1_ values reduce the 3 dB bandwidth but also introduce increased oscillations in the circuit. The rise time at higher resistances was approximately 20 µs. However, due to the small capacitance of the piezoelectric component, large inductance is required, in the range of 10 H to shift the cut-off frequency towards lower values. Moreover, the parallel resistor needs to have a reasonably high value to reduce the oscillations in the circuit.

**Figure 6:**
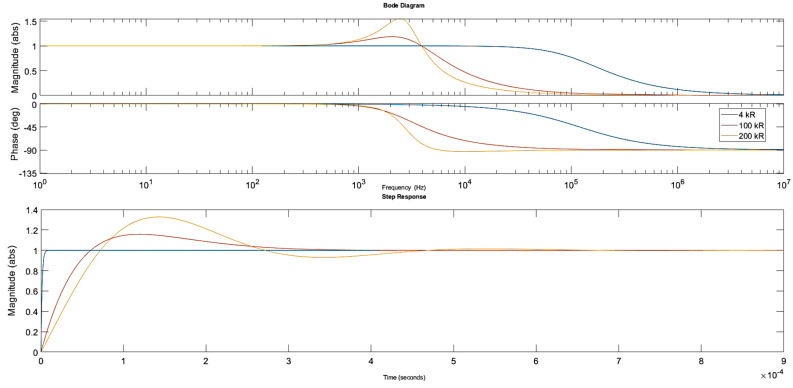
Bode and step response plots for the different parallel resistance values. The inductor (10 H, 3.5 kΩ) and the piezo stack settings remained constant.

The simulation results shown in Figure 6 are for 10 H inductance and for the following settings of the parallel resistance: 4 kΩ, 100 kΩ, 200 kΩ. The piezoelectric component parameters remained unchanged.

Clearly, the 3 dB bandwidth, slew rate and the oscillations were significantly reduced. The rise time is approximately 60 µs for the 200 kΩ parallel resistance settings and the bandwidth is nearly 5 kHz, sufficient to meet IEC harmonic measurement specifications. Consequently, the 10 H inductor and a 150 kΩ parallel resistor were preselected as optimal components for the analysed circuit with the rise time of approximately 52 µs and the 3 dB bandwidth of 5.3 kHz.

### 2.5. Lightning Waveform Simulations

Apart from the Bode analysis described above, the transient analysis of the circuit response to the 1.2/50 µs waveform that was used to emulate a lightning waveform was also performed for the component sets described in the previous section. 

**Figure 7:**
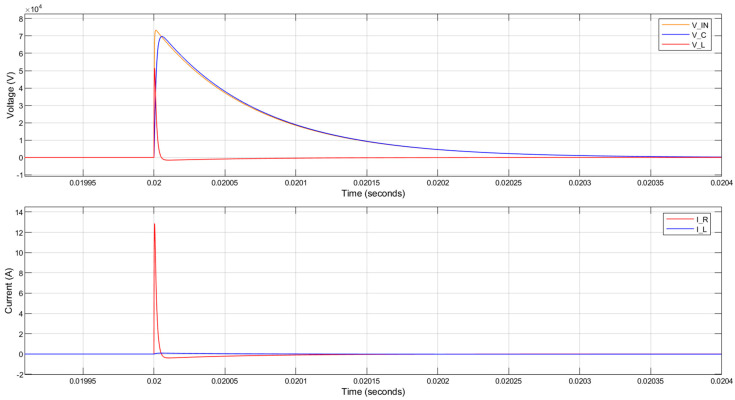
MATLAB simulation results for the circuit response to a lightning impulse with 75 kV peak voltage. The parallel resistance is 4 kΩ. (V_IN—input 1.2/50 µs lightning impulse; V_C—voltage across the piezoelectric component; V_L—voltage across the inductor; I_R—current through the parallel resistor; I_L—current through the inductor).

When R_1_ = 4 kΩ is used with a 1 H inductor, the 3 dB bandwidth and the rise time are not reduced sufficiently to attenuate the lightning impulse applied to the circuit. The voltage across the piezoelectric component was almost the same as the input 1.2/50 µs lightning impulse, as can be seen in Figure 7. The current through the parallel resistor is over 12 A peak, which is also undesirable.

**Figure 8:**
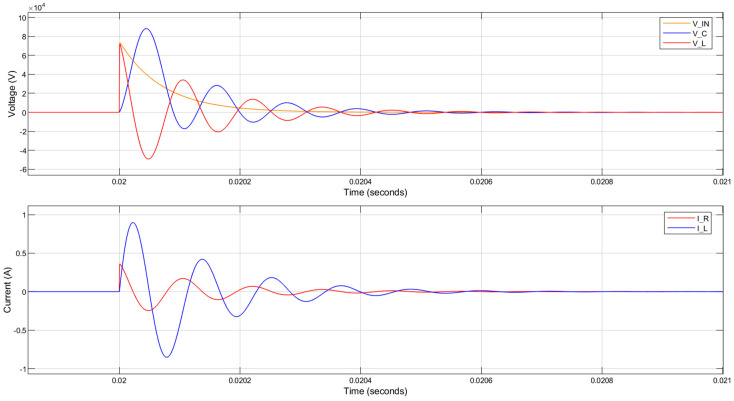
MATLAB simulation results for the circuit response to a lightning impulse with 75 kV peak voltage. The inductance is 1 H and the parallel resistance is 200 kΩ.

The circuit response when a 1 H inductor is used with a 200 kΩ parallel resistance is shown in Figure 8. Although the voltage across the piezoelectric component (V_C) was slowed down in comparison to the input 1.2/50 µs lightning impulse (V_IN), there are large oscillations generated and the peak voltage across the piezoelectric component is even greater than the magnitude of the applied lightning impulse. This is undesirable even though the currents in the circuit are significantly reduced.

**Figure 9:**
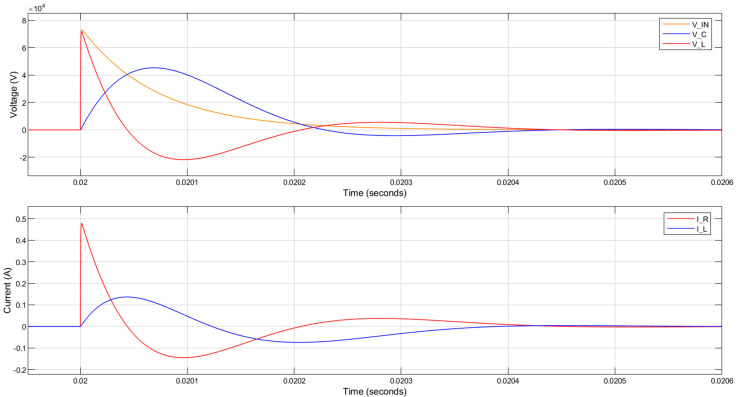
MATLAB simulation results for circuit response to a lightning impulse with 75 kV peak voltage. The inductance is 10 H and the parallel resistance is 150 kΩ.

The circuit response when a 10 H inductor is used with a 150 kΩ parallel resistance is shown in Figure 9. The voltage across the piezoelectric component (V_C) is reduced to 60% of the lightning impulse voltage (V_IN) and the rise time is decreased nearly 60 times. The peak current flowing through the parallel resistor (I_R) and the inductor (I_L) are below 0.5 A and 0.2 A, respectively. 

The simulation results presented above were used for finite element analysis discussed below.

### 2.6. Finite Element Analysis

**Figure 10:**
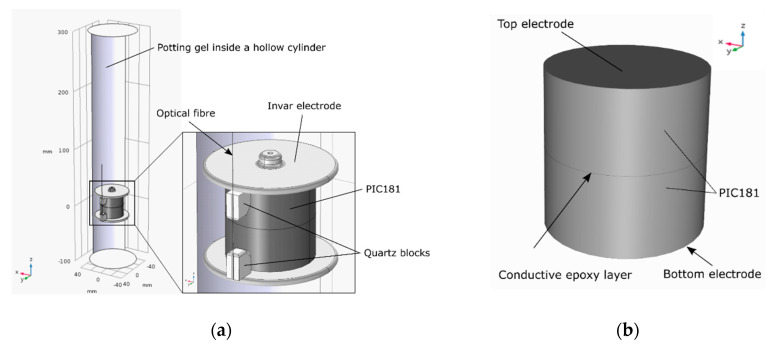
MV PVT inner components: (**a**) general view; and (**b**) simplified transducer geometry implemented in COMSOL Multiphysics^®^.

To calculate the stress generated in the material during lightning impulse events, the finite element analysis (FEA) was performed in COMSOL Multiphysics^®^ software. A general view of the MV PVT inner components is shown in Figure 10a and a simplified model with only the PIC181 geometry that was analysed in COMSOL is shown in Figure 10b.

The piezoelectric component was formed by stacking two 20 mm-thick cylinders with a 40 mm diameter. In the sensor, the cylinders were connected together with a very thin conductive epoxy layer, which in the model was simulated as a floating potential. The top electrode of the weight of 0.07 kg was included in the model under the ‘Added Mass’ node in solid mechanics. Since the piezoelectric stack was attached to the top and bottom electrodes with the conductive epoxy mentioned earlier, it was assumed that there was no movement of the stack surface in the radial direction of the electrodes. The top electrode was treated as a rigid connector so the stack could move only in the z direction. The stack was also fixed at the bottom electrode meaning that the surface touching the bottom electrode could not move in any direction. The gravity was added to all components. The bottom electrode was electrically grounded while the top electrode was connected to a terminal voltage. 

**Figure 11:**
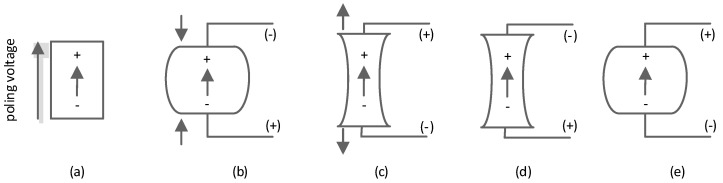
Reaction of a piezoelectric component to applied force or voltage [16]: poling direction (**a**), compression applied to the material in the poling direction generates voltage of the same polarity as the poling voltage (**b**); tension applied to the material in the poling direction generates voltage of the opposite polarity to the poling voltage (**c**); voltage of the same polarity as the poling voltage causes the material extension in the direction of the poling voltage (**d**); voltage of the opposite polarity as the poling voltage causes the material contraction in the direction of the poling voltage (**e**).

The poling direction for the component was replicated from the real component and aligned with the z positive direction. This means that when the material was compressed in the z direction, the voltage on the top electrode was negative (Figure 11b). For negative voltages applied to the top electrode, the material will be tensioned as shown in Figure 11d.

Some of the PIC181 specifications are summarized in Table 4.

A number of simulations were run in time domain studies to evaluate the expected response of the stack to a nominal sinusoidal 50 Hz voltage and to the considered lightning impulse events. 

**Figure 12:**
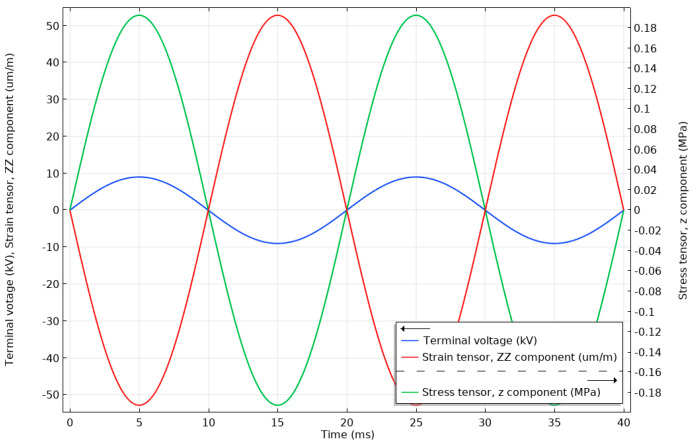
Instantaneous terminal voltage, stress and strain in the stack when subjected to a nominal 6.35 kV (RMS) voltage. Stress and strain are measured on the surface of the stack in the middle of its thickness.

The instantaneous stress and strain on the stack surface in the middle of its thickness when a PVT nominal voltage of 6.35 kV (9 kV peak) was applied is shown in Figure 12. As can be seen, the stress in the material was below 2% of the 10 MPa tensile limit. 

**Figure 13:**
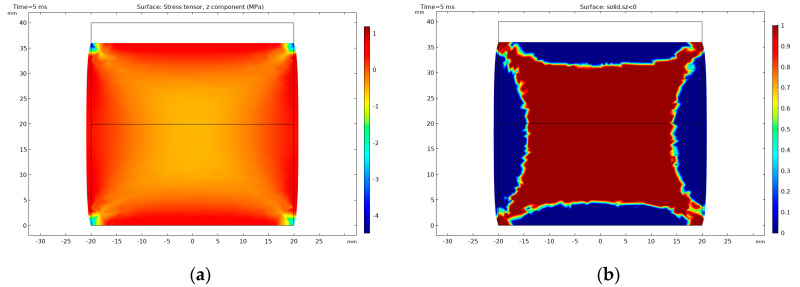
Cut plane through the centre of the stack: (**a**) stress distribution in the material is shown at a positive voltage of 9 kV; (**b**) compressive (red) and tensile (blue) stress regions in the stack at 9 kV.

When a positive voltage is applied to the piezoelectric disc, it will contract in its axial direction and will broaden in the diameter. It can be clearly seen in Figure 13 where a positive voltage of 9 kV was applied to the PIC181 component. The stress is not evenly distributed in the material, and the stress tensor z-component shows tension on the surface of the stack and compression inside the component in MPa, as can be seen in Figure 13a. It can be easier distinguished in Figure 13b where the compressive stress region is marked in red and the tensile stress region is marked in blue. The non-uniform stress distribution is due to the fixing of the stack and its inertia. 

Similar settings for the model were kept during the lightning impulse simulations. Firstly, the PZT stack was subjected to positive and negative 1.2/50 µs impulses with 75 kV and 60 kV peak voltage amplitudes as per the relevant standard requirements, and the stress and strain levels on the surface of the material were analysed. The second consideration involved the attenuated lightning impulse signal expected on the piezoelectric component as determined from the MATLAB simulations discussed in Section 2.5. Additionally, in this case, the stress and strain levels on the surface of the material were analysed.

**Figure 14:**
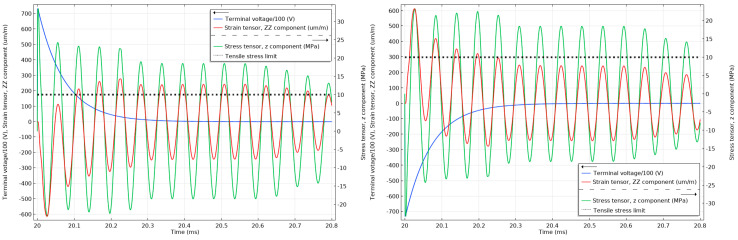
Stress and strain in the undamped stack when subjected to 1.2/50 µs 75 kV positive (**left**) and negative lightning impulses (**right**). The tensile stress limit of 10 MPa is marked by a black dotted line (the terminal voltage is the voltage applied to the top electrode of the stack.).

**Figure 15:**
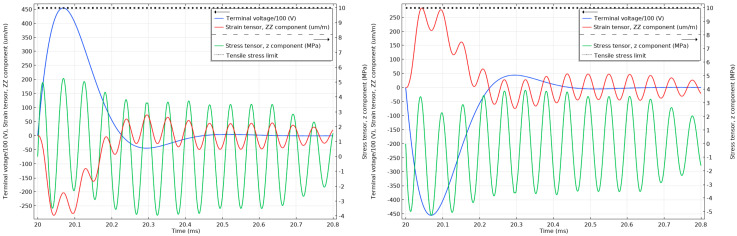
Stress and strain in the undamped stack when subjected to attenuated 75 kV positive (**left**) and negative (**right**) lightning impulses. The tensile stress limit of 10 MPa is marked by a black dotted line (the terminal voltage is the voltage applied to the top electrode of the stack.).

**Figure 16:**
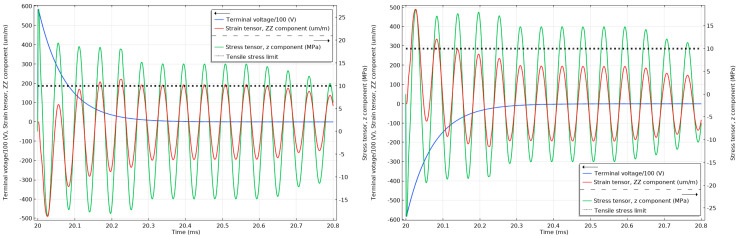
Stress and strain in the undamped stack when subjected to 1.2/50 µs 60 kV positive (**left**) and negative lightning impulses (**right**). The tensile stress limit of 10 MPa is marked by a black dotted line (the terminal voltage is the voltage applied to the top electrode of the stack.)

**Figure 17:**
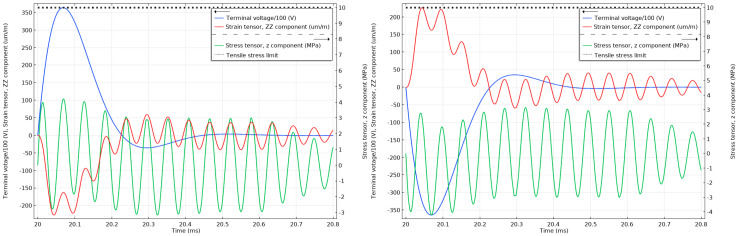
Stress and strain in the undamped stack when subjected to attenuated 60 kV positive (**left**) and negative (**right**) lightning impulses. The tensile stress limit of 10 MPa is marked by a black dotted line. (The terminal voltage is the voltage applied to the top electrode of the stack.).

As can be seen in Figure 14, the strain cannot react instantly to the electric impulse of 75 kV, and until the strain has reacted, the material will respond with an opposite stress to compensate. The stress in the material is above the 10 MPa tensile limit (with the maximum peak reaching 35 MPa) for the unattenuated 75 kV lightning impulses and below 50% of the 10 MPa tensile limit when the impulses are attenuated, as can be seen in Figure 15. Similar effects can be observed in Figure 16 when the unattenuated 60 kV impulses are applied to the material, and in Figure 17 when the 60 kV impulses are attenuated.

**Figure 18:**
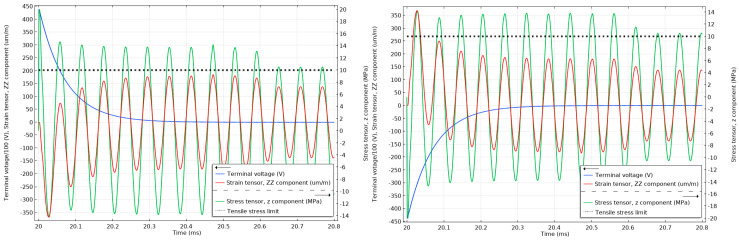
Stress and strain in the undamped stack when subjected to 1.2/50 µs 45 kV positive (**left**) and negative lightning impulses (**right**). The tensile stress limit of 10 MPa is marked by a black dotted line. (The terminal voltage is the voltage applied to the top electrode of the stack.).

For comparison, the stress in the components when subjected to positive and negative 1.2/50 us impulses with 45 kV peak voltage amplitude are shown in Figure 18. This is the case, when an alternative approach of protecting the PVT with overvoltage spark gaps is applied. Since the PVT would need to withstand a power frequency voltage of 28 kV (40 kV peak), the spark gap overvoltage limit would need to be at least 45 kV. Clearly, the 10 MPa tensile limit was breached again.

Based on the above analysis, it was concluded that the proposed solution can protect the component from permanent damage, and thus, a prototype lighting impulse attenuator was constructed as described in the following section.

### 2.7. Lightning Impulse Attenuator Construction

**Figure 19:**
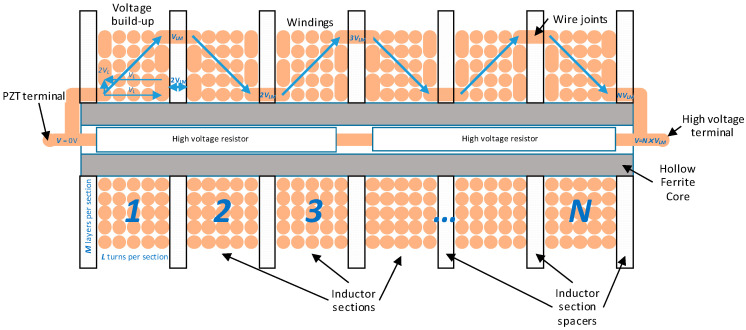
Schematic diagram of the attenuator electrical connections (winding diameter and the components are not drawn to scale).

To achieve an inductance of 10 H, a multilayer ferrite-core inductor was proposed. Since the inductor needs to withstand the lightning impulse with a peak voltage of 75 kV, it is required to wind the inductor in a special way to minimize the wire-to-wire voltage and to prevent any dielectric breakdown between the inductor windings (see Figure 19 for details). Therefore, it was proposed to divide the inductor winding into 10 subsections taking into account the available space limitations for the coil in the MV insulator. The available space in the insulator was assumed to be limited to a cylinder with a height of 300 mm and a diameter of 600 mm to allow for connections between the PVT and the top flange of the insulator and to provide enough clearance for potting with a dielectric gel. It was assumed that each section of the coil should have an inductance of approximately 1 H and the voltage across each section should be limited to 7.5 kV. To further increase the coil inductance without amending its dimensions, a ferrite core was used. It is also desirable to ensure the symmetry of the electrical field distribution around the attenuator when placed in the MV insulator and connected to the PVT. Therefore, it was determined that the use of a hollow cylinder-shaped core would be the most appropriate allowing for placing the required resistors in the centre of the core.

Consequently, the lightning impulse attenuator consists of three main components:

1. A coil wound on a dedicated former;
2. A ferromagnetic core made of ferrite toroids;
3. High voltage (HV) resistors connected in parallel with the coil.

#### 2.7.1. Attenuator Design

Based on the MATLAB simulations presented in Section 2.5, AWG32 wire should be sufficient to carry a 0.2 A peak current during lightning events without any overheating or damage to the wire. To accommodate the required resistors and the ferrite core (described in more details in the next subsection), it was assumed that the inner diameter of the inductor should be approximately 30 mm. 

A single section inductance was initially calculated based on the Wheeler’s approximation that is accurate to <1% for air-core inductor coils with square shaped cross-sections [17,18]:

(2) $$L\left(\mu H\right)\;=\;\frac{31.6n^{2}r_{1}^{2}}{6r_{1}^{2}\;+\;9l\;+\;10\left(r_{2}\;-\;r_{1}\right)}$$

where 

*L(µH)*—inductance in µH;

*n*—total number of turns;

*r*_1_ and *r*_2_—inner and outer radius of the coil in meters;

*l*—length of the coil in meters. 

To achieve the required inductance of 1 H, a single section of the coil should have approximately 5200 turns distributed in 46 layers with 112 turns per layer. By connecting 10 such sections in series, the total coil inductance should be around 10 H with the winding resistance of around 3.5 kΩ. 

It should be noted that the inductance of 10 H is not a strict but rather a minimum requirement for the attenuator. If higher value can be achieved, the even greater attenuation of the lightning impulse can be expected. However, care must be taken not to reduce the sensor bandwidth to the point that the accuracy requirements set by the standard for the relevant harmonics are not met. An increase in the coil inductance can be achieved without any changes to the inductor dimensions by using a high permeability ferromagnetic core insert. 

#### 2.7.2. Inductor Construction

To prevent the coil from the dielectric breakdown between the wires, the coil requires a special way of winding. The winding needs to be divided into subsections and the interconnections between the sections need to be done as shown in Figure 19. In the diagram shown in Figure 19, *L* is the number of winding turns per section, *M* is the number of winding layers per section, and *N* is the number of sections. Winding separation defined by the wire enamel layer must prevent electrical discharge when exposed to the voltage differential of 2*V_L_* (*V_L_* is the voltage drop along one winding layer). Therefore, the inductor section spacers (or partitions) must prevent electrical discharge when exposed to the voltage differential of 2*V_LM_* (*V_LM_* is the voltage built up within one section). Ideally, the spacer’s dielectric constant should match that of the wire enamel and dielectric filler (e.g., resin) to prevent high electric field gradients.

Assuming ten 1 H sections of the inductor (*N* = 10), the 75 kV impulse peak voltage was spread across the sections with a voltage of 7.5 kV per section (*V_LM_* = 7.5 kV). At 46 layers, voltage across a single layer was approximately 160 V, and *2V_L_* was around 320 V. The partitions between the sections must have a breakdown voltage higher than 14 kV.

Dielectric filler (e.g., resin or dielectric gel) with dielectric constant matching that of wire enamel must be used to displace the air between the windings to prevent high electric field gradients that otherwise could lead to dielectric breakdown in the inductor.

Consequently, a prototype inductor was wound with a 0.2 mm Grade 2 (dual coat) enamelled wire on the polylactic acid (PLA) coil former. The wire had a breakdown voltage of 7 kV. The PLA was chosen as a prototype material that can be easily 3D printed and has a sufficient breakdown strength (~ 4 MV/cm [16]). The PLA coil former was designed to hold the core and the resistors inside, while the coil windings could be placed in dedicated sections outside the core and the resistors. Special groves and holes in the former partitions were made to allow for the windings interconnections as described above. Each section of the coil contained 5200 turns. The coil was covered with GL96 tape on the outside, which stuck over the coil to hold the windings tight. The assembly was secured with a varnish coat on the inside of the sections and over the taped coil to provide additional mechanical strength.

The ferromagnetic core was made of 27 ferrite toroids which were epoxied together to form a 27 cm-long hollow cylinder. The toroids were made of a high permeability (µ = 10,000) Material W (ZW-42508-TC (Epoxy coating) from Magnetics [19]) having a thickness of approximately 10 mm and inner and outer diameter (ID/OD) of approximately 15/25 mm. The epoxy coating on the toroids is suitable for continuous operation at 200 °C and have the minimum breakdown voltage rating of 2 kV wire-to-wire. The assembled core was secured by a heat shrink with an additional internal epoxy layer, and its outer diameter was below 30 mm so that it could be inserted into the coil PLA former with a sufficient clearance to be potted with a dielectric gel later on.

**Figure 20:**
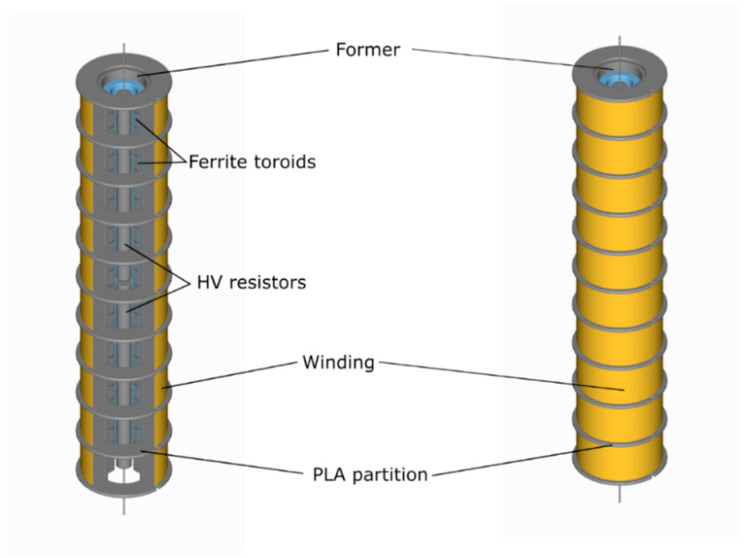
Lightning impulse attenuator assembly.

To ensure the symmetric electrical field distribution around the PVT component and the attenuator, two 75 kΩ HV resistors were connected in series to provide a total resistance of 150 kΩ and placed inside the ferrite toroids as shown in Figure 20. They were then connected in parallel with the coil windings. The measured air-cored inductance value was 13.95 H and 18.40 H with the ferrite core. The resistance of the coil was 3.65 kΩ.

**Figure 21:**
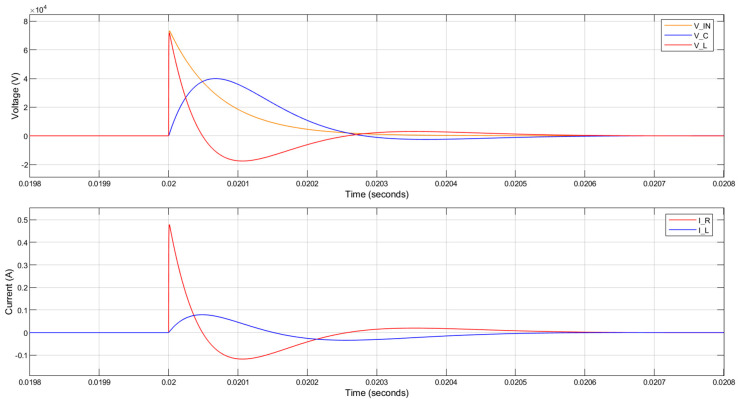
MATLAB simulation results for the circuit response to a lightning impulse with 75 kV peak voltage. The coil inductance is 18.4 H, its resistance is 3.65 kΩ and the parallel resistance is 150 kΩ.

As shown in Figure 21, when these parameters are set in the MATLAB simulation described in Section 5, the peak voltage across the piezoelectric component (V_C) was expected to be reduced to approximately 40 kV when a 75 kV lightning impulse is applied to the PVT terminals. Consequently, the tensile stresses in the material should be well below the 10 MPa limit discussed earlier. Furthermore, the peak current flowing through the coil (I_L) should be below 0.1 A.

**Figure 22:**
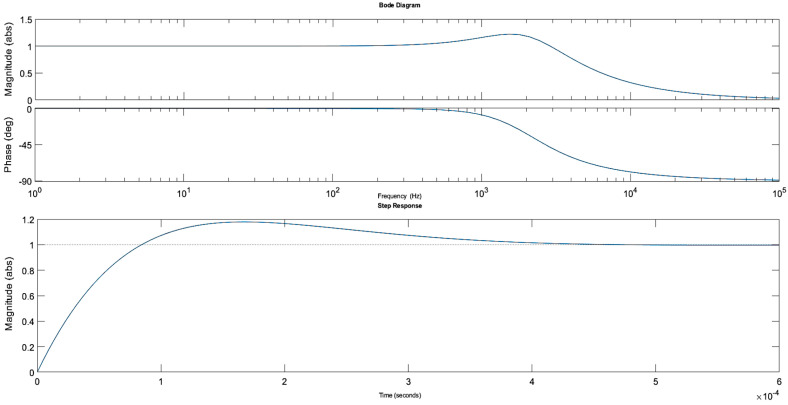
Bode and step response plots for the final lightning impulse attenuator. The coil inductance is 18.4 H, its resistance is 3.65 kΩ and the parallel resistance is 150 kΩ.

For the settings specified above, the Bode and rise time plots simulated in MATLAB are shown Figure 22. The rise time was approximately 63 µs and the 3 dB bandwidth was around 4.5 kHz. The expected phase shift at 50 Hz was −0.024 degrees. 

#### 2.7.3. PVT Assembly

**Figure 23:**
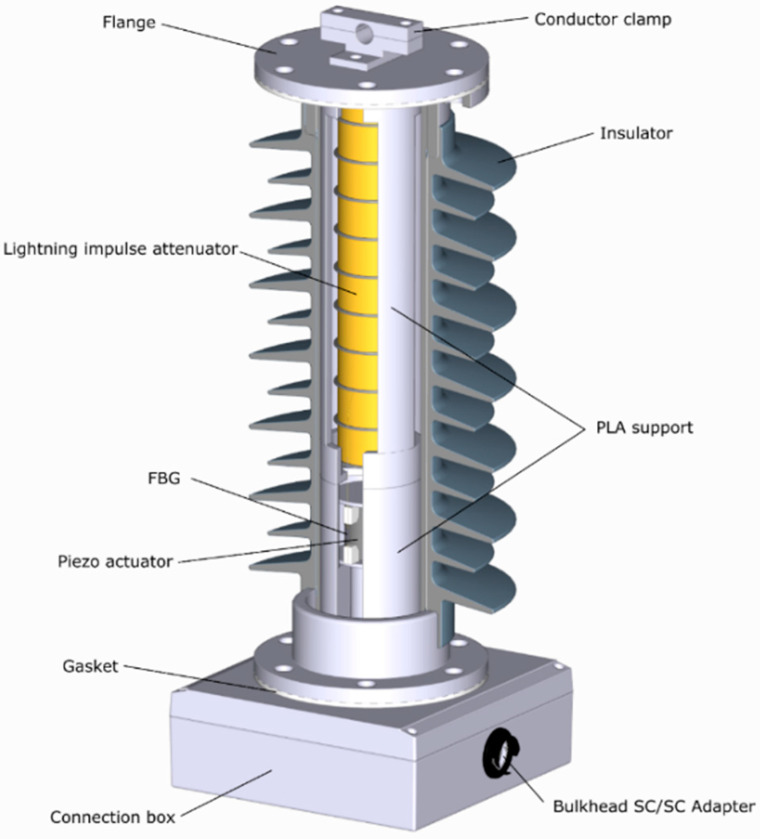
MV PVT improved design.

To hold the lightning impulse attenuator securely in the insulator and to facilitate a more straightforward sensor assembly process, a dedicated PLA support was designed and fabricated allowing to stack the PVT sensor and the attenuator inside the MV insulator as shown in Figure 23. This arrangement preserved the symmetry of the electric field distribution around the components that were potted in a dielectric gel. The electrical connections were made between the bottom (grounded) flange of the insulator and the bottom electrode of the PVT. The top electrode of the PVT was connected to the bottom connector of the attenuator, and its opposite connector was connected to the top flange of the insulator. The entire assembly of the revised design of the MV PVT is shown in Figure 23.

#### 2.7.4. Lightning Impulse Tests

As indicated in Section 2.1, since the PVT was designed for an 11 kV network, it is classified as a device with the highest voltage for the equipment of 12 kV. As per IEC 60044-7 [5], the required lightning impulse withstand voltage level should be either 60 or 75 kV peak and the full-wave impulse should have the front time of 1.2 µs (±30% tolerance) and the time to half-value of 50 µs (±20% tolerance) as defined by IEC 60060-1 [20]. The test procedure requires the application of 15 consecutive impulses of both positive and negative polarities to the device under test (DUT). The DUT is deemed to have passed the tests if there are no disruptive discharges or flashovers, or if no more than two flashovers for each polarity occur, which was confirmed by five consecutive impulse withstands following the last disruptive discharge.

**Figure 24:**
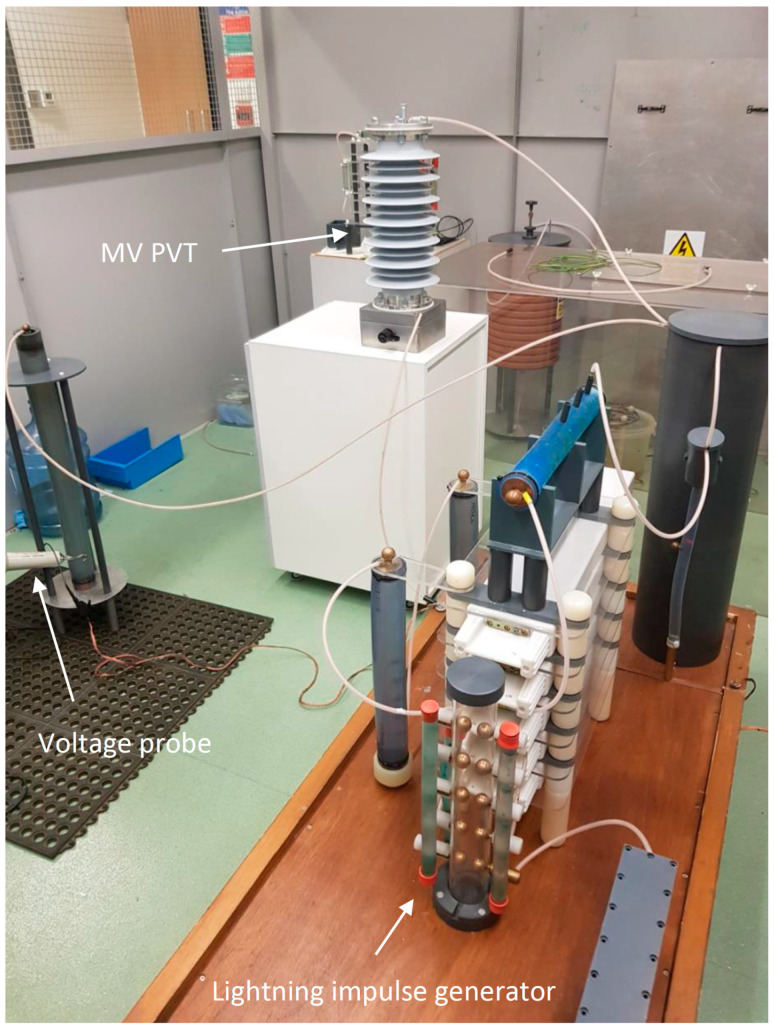
Lightning impulse voltage tests.

To validate the MV PVT performance against the lightning impulse withstand voltage, the tests were carried out by a subcontractor (Samtech Ltd., Glasgow, United Kingdom), according to the relevant IEC standards. During the lightning impulse tests, the MV PVT was connected to a three-stage Marx lightning impulse generator, as shown in Figure 24. The generator was capable of generating the required impulses with the front time and the time to half-value of 1.24 µs and 50.2 µs, respectively, for the positive and negative polarity impulses, satisfying the requirements set by the IEC standards.

Accordingly, the prototype sensor was subjected to 60 kV lightning impulses of both positive and negative polarities. The device successfully endured all the impulses without any disruptive discharges or voltage collapses. 

## 3. Sensor Calibration and Accuracy Testing

### 3.1. Experimental Setup

**Figure 25:**
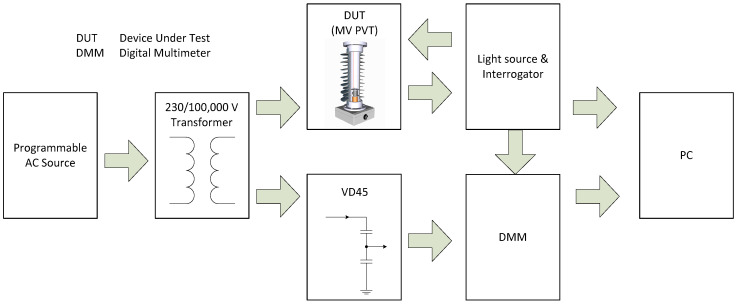
Experimental setup for MV voltage sensors calibration and testing.

The MV PVT calibration was performed using a comparison method specified by IEC 60060-2 [21]. The DUT was connected in parallel with a reference measuring system, traceable to a National Metrology Institute, and calibrated by the comparison of both measurement system outputs. The reference measuring system was formed by a Ross Engineering C-divider VD45 suitable for measuring DC and AC voltages up to 45 kV and Keysight 3458A (UKAS certified). The measurements were performed simultaneously on both measurement systems, and the digital multimeter (DMM) was synchronized with the optical interrogation system. A diagram of the experimental setup is shown in Figure 25. 

The tested sensor was illuminated by a broadband light source and the reflected signals were analysed using an FBG interrogator, I-MON 256 USB, connected to a PC. The test voltage was provided from a step-up transformer capable of delivering up to 100 kV. The transformer was powered from a programmable AC source, Chroma 61512, capable of delivering 18 kVA at 300 V. The voltage reference signal was obtained from the VD45 providing a nominal 4035:1 voltage division ratio. The VD45 output was monitored by a Keysight 3458A DMM connected to a PC via USB/GPIB cable and isolated from a PC via an optical USB cable. The optical system clock (4 kHz) was used to synchronize the interrogator readings with the DMM readings. The clock signal from I-MON was provided to the DMM via optical link. Unfortunately, in the current system implementation, the provision of an additional trigger that would release both systems at the same time was not possible. Therefore, the sensor phase calibration was not possible and only amplitude calibration was performed. 

### 3.2. Sensor Calibration and Accuracy Testing

To calibrate the MV PVT, a 50 Hz sinusoidal voltage was applied to the sensor in the range from 2% to 120% of the nominal voltage (6.35 kV) in 10% steps between 10% and 120% of the nominal. The calibration and testing of the sensor were performed at room temperature (21 ± 1 °C). The readings of the optical signals from the sensor and the readings from the DMM were recorded together at each voltage level. As described above, even though the samples from both systems were acquired at the same time and synchronized, signal acquisition started at different moments each time the software controlling acquisition was started, causing a random phase displacement between the acquired signals.

At each voltage level, 8000 samples, equivalent to 100 periods of 50 Hz signals, were captured. The rms values were then calculated from 10 periods for 10 measurements at each voltage level. The measurements were then repeated three times.

**Figure 26:**
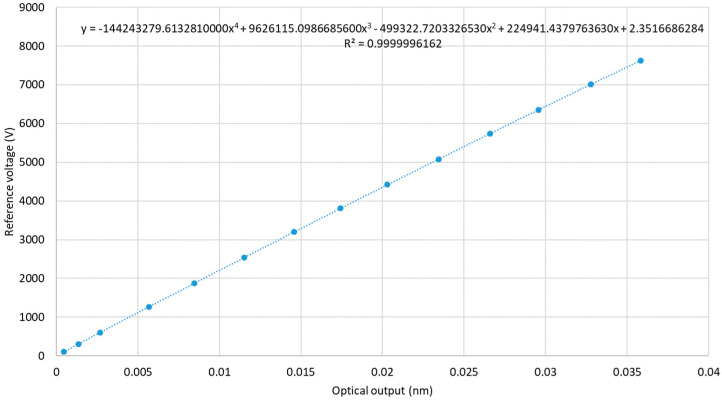
MV PVT calibration curve.

The RMS calculations were performed for the main frequency components of the signals using FFT analysis. The sensor characteristic, shown in Figure 26, was then obtained by averaging 10 measurements at each voltage level as per IEC60060-2 [21]. To convert the DMM output voltage (0–10 V range) to the primary voltage, a value of 4280 was assumed for the VD45 scale factor based on the available VD45 calibration data.

To create the sensor calibration curve, shown in Figure 26, a fourth order polynomial was fitted into the data obtained from the first characterization run with a 95% confidence level. The errors due to the goodness of fit and the associated uncertainties were below 0.0005, two orders of magnitude lower than the uncertainties associated with the VD45 stability and scale factor estimation which were ±0.02 for the extended uncertainty with a 95% confidence level. Therefore, they can be neglected.

### 3.3. Accuracy Testing

To assess the suitability of the sensor for protection and metering purposes, 50 Hz voltage waveforms with amplitudes of 2%, 5% and between 80% and 120% of the device rated voltage were applied as part of the test procedure. For each case, the measurements were repeated three times. 

The amplitude errors were calculated according to the following equation:

(3) $$\varepsilon \left(\%\right)\;=\frac{V_{p}\;-\;V_{rec}}{V_{p}}\cdot 100$$

where *V_p_* is the rms value of the primary voltage and *V_rec_* is the rms value of the reconstructed voltage.

**Figure 27:**
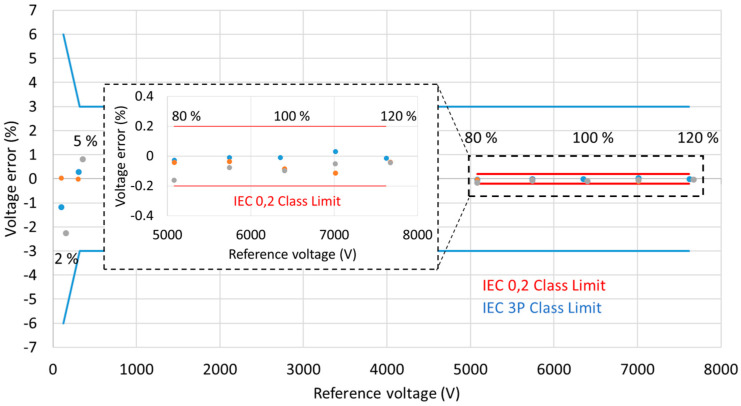
MV PVT voltage errors. The error limits for the 0.2 metering class are marked by the red lines for the range of 80–120% of the sensor rated voltage (6.35 kV). The 3P protection class limits are marked by the blue lines.

The voltage errors for the three consecutive test runs are shown in Figure 27. As indicated in Section 2, for the 0,2 metering class devices, the voltage errors at the rated frequency should not exceed 0.2% at any voltage between 80% and 120% of the rated voltage. This is indicated in the figure by the red lines. Clearly, the MV PVT voltage error is within those limits. 

For the 3P protection class devices, voltage errors at 5% of the nominal voltage and at the rated voltage factor (120% for the sensor) should be below 3%; at 2% of the nominal voltage, the errors should be below 6%. Again, the MV PVT clearly meets this level of performance, with errors below 2.5% at 2% of the nominal voltage, below 1% at 5% of the nominal voltage and below 0.2% at 120% of the nominal voltage. 

Thus, the same device has the potential to meet the 3P protection class and 0.2 metering class requirements.

## 4. Discussion

As indicated in the Introduction section, the accuracy and safety requirements for the proposed device were based on IEC 60044-7 for Electronic Voltage Transformers as the most appropriate standard. Although the IEC 60044 standard is being replaced by the new IEC 61869 standard, the equivalent part 7 of the new standard is not available at the time of writing of this paper, and the document forecast publication date is currently set to December 2022 [22]. Until then, IEC 60044-7 is still in force.

As indicated in Section 3.1, the sensor phase calibration, and hence, the phase errors’ estimation was not possible due to the current implementation of the sensor calibration system. In the current system implementation, the provision of an additional trigger that would release both systems at the same time was not possible. While data samples from both systems can be acquired at the same time and synchronized, the acquisition of the sensor and reference signals starts at different moments each time the software controlling acquisition is started, causing a random phase displacement between the acquired signals. However, based on the Bode analysis of the PVT circuitry (discussed in Section 2.7.3.), the expected phase displacement between the primary voltage and the voltage across the sensor is below 2 minutes, thus having the potential to meet the 0,2 and 3 P class requirements. The phase errors will be confirmed experimentally as soon as the improved calibration system is available in the future.

It should also be noted that the scale factor (voltage ratio) and phase displacement of the VD45 voltage reference may change with voltage, frequency, temperature and time. The divider was initially characterized by the National Metrology Institute, and the PVT calibration and tests were carried out within a few hours on a single day. Although the divider was sufficiently stable over that period, further work is required to establish its performance in terms of long-term stability.

## 5. Conclusions

In this paper, the design, construction and characterization of a photonic voltage transducer for the application of the distributed sensing of voltage on medium-voltage networks (11 kV) was presented. The device was equipped with a novel lightning impulse protection circuitry, enabling it to satisfy the safety requirements applicable to 11 kV networks. Accordingly, the sensor successfully passed the lightning impulse withstand tests as specified by the IEC 60044-7 standard. The accuracy performance was also evaluated against the IEC 60044-7 standard. The preliminary results of the accuracy tests showed that the sensor has the potential to meet the accuracy requirements of the IEC 0,2 class for metering devices and 3P class for protective devices. 

Future work will focus on performing additional tests, such as temperature, routine and special tests according to the relevant standards and verifying the sensor phase errors and long-term performance characteristics.

## 6. Patents

Pawel Niewczas and Grzegorz Fusiek: Photonic Voltage Transducer (Overvoltage Protector), GB1919237.6, 2019

## Figures and Tables

**Table 1:** Voltage withstand requirements for electronic voltage transformers with primary terminals having the highest voltage for equipment below 300 kV [5].

Primary Rated Voltage (kV)	Voltage Factor 1.2 (kV)	Voltage Factor 1.5 (kV)	Highest Voltage for Equipment (rms) (kV)	Rated Power-Frequency withstand Voltage (rms) (kV)	Rated Lightning-Impulse withstand Voltage (peak) (kV)
6.35	7.6	9.5	12	28	60 or 75

**Table 2:** Protection class accuracy requirements [5]. In the table, *U*_p_ and *U*_pn_ are the primary voltage and nominal (rated) primary voltage, respectively; ε_u_ and φ_e_ are the voltage (ratio) and phase errors, respectively.

Accuracy Class	*U*_p_/*U*_pn_								
	2			5			X ^(1)^		
	ε_u_ % ±	φ_e_ Minutes ±	φ_e_ Centiradians ±	ε_u_ % ±	φ_e_ Minutes ±	φ_e_ Centiradians ±	ε_u_ % ±	φ_e_ Minutes ±	φ_e_ Centiradians ±
3P	6	240	7	3	120	3.5	3	120	3.5
6P	12	480	14	6	240	7	6	240	7

^(1)^ x is the rated voltage factor multiplied by 100.

**Table 3:** Metering class accuracy requirements [5].

Accuracy Class	ε_u_ Percentage Voltage (Ratio) Error ±	φ_e_ Phase Error ±	
		Minutes	Centiradians
0.1	0.1	5	0.15
0.2	0.2	10	0.3
0.5	0.5	20	0.6
1.0	1.0	40	1.2
3.0	3.0	Not specified	

**Table 4:** Specifications of the PIC181 piezoelectric material utilized for the MV PVT device.

Cross-Sectional Area (mm^2^)	1256
Thickness (mm)	40
Piezoelectric charge constant d33 (pm/V)	265
Resistance Rp (MΩ)	200
Capacitance Cp (nF)	0.33
Strain-to-voltage sensitivity (nε/V)	13.3
Maximum permissible electric field strength of the material (kV/mm)	2.5
Maximum compressive stress (MPa)	100
Maximum tensile stress (MPa)	10

## Dataset

All data underpinning this publication are openly available from the University of Strathclyde KnowledgeBase at https://doi.org/10.15129/03b5240d-4392-4a87-9e51-7f9f33fd17cc.

## Abbreviations

The following abbreviations are used in this manuscript:

DMM	digital multimeter
DUT	device under test
FBG	fiber Bragg grating
GPIB	general purpose interface bus
HV	high voltage
ID/OD	inner diameter/outer diameter
IEC	International Electrotechnical Commission
MV	medium voltage
PC	personal computer
PEEK	polyether ether ketone
PVT	photonic voltage transducer
RMS	root mean square
USB	universal serial bus
VD	voltage divider
